# Refeeding syndrome influences outcome of anorexia nervosa patients in intensive care unit: an observational study

**DOI:** 10.1186/cc9274

**Published:** 2010-09-28

**Authors:** Marie Vignaud, Jean-Michel Constantin, Marc Ruivard, Michele Villemeyre-Plane, Emmanuel Futier, Jean-Etienne Bazin, Djillali Annane

**Affiliations:** 1 General ICU, Estaing Hospital, University Hospital of Clermont-Ferrand, 1 Place Lucie Aubrac, 63000 Clermont-Ferrand, France; 2 Auvergne Anoria Network, University Hospital of Clermont-Ferrand, 1 Place Lucie Aubrac, 63000 Clermont-Ferrand, France; 3 Internal medicine department, Estaing Hospital, University Hospital of Clermont-Ferrand, 1 Place Lucie Aubrac, 63000 Clermont-Ferrand, France; 4 Centre medico-psychiatrique B, University Hospital of Clermont-Ferrand, 1 Place Lucie Aubrac, 63000 Clermont-Ferrand, France; 5 General Intensive Care Unit, Raymond Poincare Hospital (AP-HP), University of Versailles, SQY, 104 Boulevard Raymond Poincare, 92380 Garches, France

## Abstract

**Introduction:**

Data on the epidemiology and management of anorexia nervosa (AN) in the intensive care unit (ICU) are scarce. The aim of this study was to evaluate the prevalence and associated morbidity and mortality of AN in French ICUs.

**Methods:**

We randomly selected 30 ICUs throughout France. Thereafter, we retrospectively analyzed all patients with AN admitted to any of these 30 ICUs between May 2006 and May 2008. We considered demographic data, diagnosis at admission and complications occurring during the stay, focusing on refeeding syndrome and management of refeeding.

**Results:**

Eleven of the 30 ICUs participated in the retrospective study, featuring 68 patients, including 62 women. Average body mass index at the admission was 12 ± 3 kg/m2. Twenty one were mechanically ventilated, mainly for neurological reasons. The reported average calorie intake was 22.3 ± 13 kcal/kg/24 h. Major diagnoses at admission were metabolic problems, refeeding survey and voluntary drug intoxication and infection. The most common complications were metabolic, hematological, hepatic, and infectious events, of which 10% occurred during refeeding. Seven patients developed refeeding syndrome. At day one, the average calorie intake was higher for patients who developed refeeding syndrome (23.2 ± 5 Kcal/kg/j; *n *= 7) versus patients without refeeding syndrome (14.1 ± 3 Kcal/kg/j; *n *= 61) *P *= 0.02. Seven patients died, two from acute respiratory distress syndrome and five from multiorgan-failure associated with major hydroelectrolytic problems.

**Conclusions:**

The frequency of AN in ICU patients is very low and the crude mortality in this group is about 10%. Prevention and early-detection of refeeding syndrome is the key point.

## Introduction

The American Psychiatric Association definition of anorexia nervosa (AN) includes refusal to maintain body weight at or above a minimum normal weight for age and size, an intense fear of gaining weight or becoming large when weight is below normal, alteration of perception of body weight or shape, and amenorrhea in postpubertal women. The disease affects 0.5% of the population and 90% of patients are women. AN has the highest mortality of any psychiatric disorder [1]. There are two types of AN. The pure restrictive form, with physical hyperactivity, accounts for 70% of patients, and the bulimic form, featuring forced vomiting, affects 30% of patients. The physiopathology of AN has not yet been fully determined, and may involve genetic, neurobiological, and environmental factors [2,3]. AN is a serious psychiatric disease with severe medical complications, including a mortality rate of 5.6% per decade from illness, 12-fold that expected for similar age- and gender-matched groups [4-6]. Hospital admission remains strongly correlated with poor outcome [7]. Brief hospital admission to an acute medical ward or an intensive care unit (ICU) at times of life-threatening crises, or after weight-loss or drug overdose, may reduce mortality [8]. However, data on the epidemiology and management of AN in the ICU are scarce. The aim of this study was to evaluate frequency of anorexic patients admitted in ICU, and to evaluate complications occurring during ICU stay and patients' outcome, focusing on nutritional management.

## Materials and methods

The study protocol was approved by our local Ethics Committee, and the requirement for informed consent was waived.

### Study design

We randomly selected 30 ICUs using the CEGEDIM list of medico-surgical and medical ICUs (*n *= 360) in France. Next, we included all patients suffering from AN admitted to participating ICUs during the last two years, in an observational study.

### Patient selection and data extraction

We included all patients with AN fulfilling the criteria of the American Psychiatric Association admitted to any of the 30 participating ICUs from May 2006 to May 2008 [1]. There were no exclusion criteria.

We recorded demographic and anthropometric data on ICU admission, any relevant medical history (including age at AN diagnosis and any suicide attempts), and the reason for admission. We searched any complications occurring during an ICU stay. Anemia, leucopenia and thrombopenia were defined by blood cell count below 10 g/dL, leucocyte count below 1.4 G/L, and platelets count below 150 G/L. Coagulation disorders were defined by prothrombin rate below 60%, or ACT superior to twice the witness. Hypothyroidism was defined by TSH rate above 5 mU/ml. Acute kidney failure was diagnosed when creatinine clearance was below 60 ml/minute. Hepatitis cytolysis was defined by transaminase increasing to three times the normal. Acute lung injury was defined by PaO_2_/F_I_O_2 _ratio between 200 and 300, and acute respiratory distress syndrome by PaO_2_/F_I_O_2 _below 200, both in ventilated patients. We also recorded vital signs, any need for life-support therapy, feeding modality (route and average intake), any iatrogenic event, crude mortality, and length of ICU stay. We particularly focused on the possible existence of refeeding syndrome, defined by all adverse events occuring during nutritional rehabilitation of malnourished patients or having undergone a prolonged fast [9].

### Statistical analysis

The data were entered into a spreadsheet (Microsoft Excel within Microsoft Office 2007; Microsoft Corp., Redmond, WA, USA). Data are expressed as frequencies for nominal variables, and as means ± standard deviations (SDs) for continuous variables. Student *t *test was used for quantitave variables. A *P-*value < 0.05 was considered statistically significant.

## Results

### Retrospective study of anorexic patients

**Figure 1:**
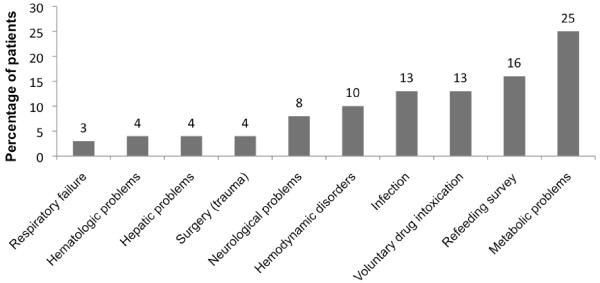
**Reasons for admission to the ICU**. The reason for admission was the main diagnosis at admission. No associated diagnosis was considered. Data are expressed as percentages of patients.

From May 2006 to May 2008, 68 patients with AN were admitted in 11 of the 30 ICUs. In 19 ICU, no AN patient were admitted in this period. Patient characteristics at baseline are shown in Table 1. The patients were predominantly female (62 patients), the mean age at the admission was 31 ± 12 years, and of very low body mass index (12 ± 3 kg/m^2^). The main reasons for admission were profound metabolic abnormalities or the need to monitor vital signs during refeeding (Figure 1). The other reasons were refeeding survey, voluntary drug intoxication, and infections. During an ICU stay, the most common complications were acute kidney failure in 19 patients (30%), and metabolic abnormalities like hypophosphatemia in 10 patients (16%) or hypokaliemia in 15 patients (24%). Hepatic dysfunction, either hepatitis cytolysis or hepatic insufficiency were found in 13 (21%) and 4 (6%) patients. Respiratory tract infections with acute lung injury and acute respiratory distress syndrome were developed in six patients (8%). Diffuse abnormal ST segment or T waves were the most common cardiac complications, reflecting repolarization problems in 10 patients (16%) (Table 2). There were seven instances of pneumothorax associated with central venous catheterization (69 catheters/61 patients). All catheters were inserted in subclavian, without the use of ultrasound for puncture guidance.

**Table 1:** Baseline characteristics of the patients

Characteristic	Data
Demographics	
Number of patients, *n*	68
Female gender, *n*	62
Age (years)	31 ± 12
Body mass index (kg/m^2^)	12 ± 3
History of anorexia nervosa	
Age at onset of illness (years)	12.7 ± 3
Antecedent suicide attempts, *n*	10
Patients receiving psychiatric treatment, *n*	33
ICU stay	
Length of stay (days)	7.6 ± 11
Tracheal intubation, *n*	21
Duration of tracheal intubation, days	5.3 ± 6
ICU admission from:	
Home, *n*	36
Medical ward, *n*	21
Psychiatric ward, *n*	10
Surgical ward, *n*	1
Destination on leaving the ICU:	
Home, *n*	8
Medical ward, *n*	42
Psychiatric ward, *n*	9
Surgical ward, *n*	2
Deceased, *n*	7

ICU: Intensive care unit

**Table 2:** Complication during ICU stay

Complication	Number of patients
Hematological	
Anemia, leukopenia, thrombopenia, *n *(%)	19 (30)
Coagulation disorders, *n *(%)	5 (7)
Endocrinological	
Hypothyroidism, *n *(%)	2 (3)
Isolated hypothermia, *n *(%)	4 (6)
Insipidus diabetes, *n *(%)	2 (3)
Neurological	
Vigilance disorders, *n *(%)	7 (10)
Agitation, *n *(%)	4 (6)
Convulsions, *n *(%)	2 (3)
Metabolic	
Acute kidney failure, *n *(%)	19 (30)
Hypophosphatemia, *n *(%)	10 (16)
Hypokaliemia, *n *(%)	15 (24)
Hyponatremia, *n *(%)	4 (6)
Metabolic alkalosis, *n *(%)	6 (8)
Metabolic acidosis, *n *(%)	3 (4)
Hypoglycemia, *n *(%)	5 (7)
Cardiovascular	
Repolarisation problems, *n *(%)	10 (16)
Bradycardia, *n *(%)	5 (7)
Thromboembolic events, *n *(%)	2 (3)
Hypotension, *n *(%)	8 (12)
Cardiac insufficiency, *n *(%)	3 (4)
Digestive	
Hepatitis cytolysis, *n *(%)	13 (21)
Hepatic insufficiency, *n *(%)	4 (6)
Acute pancreatitis, *n *(%)	2 (3)
Respiratory track infection	
Acute lung injury, *n *(%)	6 (8)
Acute respiratory distress syndrome, *n *(%)	6 (8)

ICU: Intensive care unit

During refeeding, the average calorie intake was 22.3 ± 13 kcal/kg/24 h. In 30 patients (44%), full calorie intake was initiated on the first day of refeeding. Refeeding was complicated in seven patients, including three patients with major hypophosphatemia and associated hemodynamic disorders, two patients with acute pancreatitis, one patient with cardiac arrest, and one patient with tetraplegia. At day one, the average calorie intake was higher for the patients who developed refeeding syndrome (23.2 ± 5 Kcal/kg/j; *n *= 7) versus patients without refeeding syndrome (14.1 ± 3 Kcal/kg/j; *n *= 61) *P *= 0.02. There was no difference in the average intake during ICU stay. The mortality rate was 71% (5 of 7) for patients with refeeding syndrome and 3% (2 of 61) for patients without the syndrome (*P *< 0.001). All patients with suspected refeeding syndrome required mechanical ventilation. In six of the seven patients with suspected refeeding syndrome, mechanical ventilation was initiated after refeeding commenced. Twenty-one patients required invasive mechanical ventilation. This was due to neurologic disorders in 12 patients and hypoxic respiratory failure in 9 patients. Seven patients died, two from refractory hypoxemia and five from multiple organ failure subsequent to major metabolic disorders and hepatic cytolysis following initiation of nutrition support. Prealbumin concentration was measured in 26 patients (38%).

## Discussion

The main findings of this study are that the prevalence of patients with AN in ICUs is very low and the crude mortality is about 10%. Particularly, inappropriate nutritional support was associated with a high prevalence of refeeding syndrome. On average, patients received a total calorie intake of 22 ± 13 kcal/kg/24 h.

The recent UK NICE (National Institute for Health and Clinical Excellence) guidelines suggested that calorie repletion in AN patients should be slow, and should depend on the assessed severity of refeeding syndrome risk [10]. For patients at high risk, the initial nutritional level should be approximately 10 kcal/kg/d, falling to as low as 5 kcal/kg/d in patients considered to be at extreme risk. A gradual increase in calorie intake, particularly during the first week of refeeding, in combination with regular biochemical and fluid balance monitoring, is important until a patient becomes metabolically stable.

Unsurprisingly, refeeding induced metabolic disorders and hepatic cytolysis in 10 to 20% of AN patients. The mean risk factors are AN *per se*, the classic forms of slump, and malnutrition related to chronic disease. Only a few studies have analyzed the incidence of refeeding syndrome in the ICU. In a prospective study, serum prealbumin concentration was the only biomarker predictive of the development of refeeding syndrome [11]. In the present study, prealbumin levels were recorded only in a third of the cases. In our retrospective study, full calorie intake was initiated on the first day of refeeding in about half of AN patients. In patients for whom calorie intake was gradually increased, physicians did not adequately appreciate the evidence of refeeding syndrome, as shown by biological abnormalities, in seven patients. In five patients, refeeding resulted in multi-organ failure and death, although nutrition was stopped. Refeeding syndrome can be defined as a potentially fatal shift in fluid and electrolyte levels that may occur in malnourished patients receiving artificial nutrition (whether enteral or parenteral) [12]. All of oral, enteral, and parenteral feeding routes were used in our study. Most experts agree that oral refeeding is the best approach to weight restoration. In situations in which patients refuse to eat, or in patients with extreme malnutrition, feeding via a nasogastric tube may be required [13]. If the digestive tract is functional, the enteral route is preferable to the parenteral even though parenteral nutrition can be safe and efficient [14,15].

As previously described [16], the observed prevalence of pneumothorax after central venous catheterization was six percent, approximately twice that usually observed in ICU patients [17]. To reduce the risk of this condition, we propose that an internal jugular site, and not a subclavian site, be used, with ultrasound guidance [18]. This proposal should be tempered by the infectious complications rate reported with that site [19].

The current recommendations for diagnostic investigation and monitoring in AN patients admitted to psychiatric and medical units may be inappropriate for ICU patients [20]. In our study, the high incidence of cardio vascular complications, particulary hypotension and repolarization problems, suggest that electrocardiography and echocardiography should be routinely performed at the admission of AN patients. In fact, in many publications a high incidence of occult left ventricular failure and pericardial effusion was reported in such patients [21]. In addition, improvement in cardiac function upon renutrition may be a good index of the quality of nutritional support. Metabolic disorders were the main reason for ICU admission. These disorders are the best-known metabolic complications in AN patients, and are caused by starvation or purgative practices. Profound hypoglycemia usually recurred after glucose administration, as a consequence of pathologic hyperinsulinism, and was associated with poor prognosis [22]. Hypokalemia, hyponatremia, hypomagnesemia, and metabolic alkalosis are associated with purgative practices or diuretic abuse. Hypophosphatemia was less often reported, although this is the most common sign of refeeding syndrome. As suggested, detection and correction of hypophosphatemia should be systematic at ICU admission of AN patients and before refeeding [23]. The second most common reason for ICU admission was nutritional support. When the body mass index is less than 12 kg/m², resting energy expenditure is only 60 to 65% of normal levels [13]. During refeeding, this expenditure increases significantly. Thus, it is a challenge for physicians to find a compromise between low nutritional input, with the risk of insufficient weight gain, and higher nutritional input, causing refeeding syndrome. Hemodynamic and electrocardiographic disorders were also common reasons for ICU referral. Hepatic cytolysis in AN patients was reported by 20% of physicians. Several studies and case reports have highlighted increases in serum liver enzymes in patients with AN or extreme malnutrition, whether or not associated with liver failure [24,25]. AN, and malnutrition in general, can be linked to neurological disorders such as psychomotor slowing, memory difficulties, and disorientation, that are generally reversible after renutrition [26]. Hematological disorders include leukoneutropenia, associated with bone marrow gelatinous degeneration macrocytic anemia, secondary to intra-erythrocytic ATP deficiency and thrombocytopenia [27,28]. Moreover, in patients with AN, a reduction in the contractile force of the diaphragm, and alteration in the regulation of respiratory centers, may induce respiratory failure.

Nineteen percent of patients had pneumonia and nine percent had acute respiratory distress syndrome. *In vitro *studies have suggested that starvation may be associated with altered cellular and humoral immunity [29,30]. Immune suppression during AN may also involve abnormal responses of the complement system and hypercorticism.

## Conclusions

Anorexia nervosa is an infrequent cause of ICU admission. Iatrogenia influences outcome of these young patients. Early recognition and prevention of refeeding syndrome is a key issue in ICU management of such patients.

## Key messages

• Anorexia nervosa is an infrequent cause of ICU admission.

• ICU physicians need recommendations to improve the management of anorexia nervosa patients.

• Early recognition and prevention of refeeding syndrome is a major issue.

• Prevention of iatrogenic events may decrease mortality of anorexia nervosa patients admitted in ICU.

## Abbreviations

AN: anorexia nervosa; ICU: intensive care unit

## Competing interests

The authors declare that they have no competing interests.

## Authors' contributions

MV and JMC participated in the design of the study, carried out the study and drafted the manuscript. MR, MVP, EF and JEB participated in the design of the study and data analysis. DA participated in the design of the study and helped to draft the manuscript. All authors read and approved the final manuscript.

## AnorexieRea study group

Sophie Cayot Constantin, General ICU, Estaing Hospital, University Hospital of Clermont-Ferrand, Clermont-Ferrand, France.

Renaud Guerin, General ICU, Estaing Hospital, University Hospital of Clermont-Ferrand, Clermont-Ferrand, France.

Matthieu Jabaudon, General ICU, Estaing Hospital, University Hospital of Clermont-Ferrand, Clermont-Ferrand, France.

Christian Chartier, General ICU, Estaing Hospital, University Hospital of Clermont-Ferrand, Clermont-Ferrand, France.

Sebastien Perbet, General ICU, Estaing Hospital, University Hospital of Clermont-Ferrand, Clermont-Ferrand, France.

Antoine Petit, General ICU, Estaing Hospital, University Hospital of Clermont-Ferrand, Clermont-Ferrand, France.

Samir Jaber, SAR B, Saint Eloi Hospital, university Hospital of Montpellier, Montpellier, France.

Gerald Chanques, SAR B, Saint Eloi Hospital, university Hospital of Montpellier, Montpellier, France.

Philippe Verdier, General ICU, Montlucon Hospital, Montlucon, France.

Robert Chausset, General ICU, Montlucon Hospital, Montlucon, France.

Dominique Guelon, RMC, University Hospital of Clermont-Ferrand, Clermont-Ferrand, France.

Claude Guerin, Medical ICU, La croix rousse, Lyon university Hospital, Lyon, France

Laurent Papazian, Medical ICU, APHM, Marseille, France.

Jean Paul Mira, Medical ICU, Cochin, APHP, Paris V University, France.

Bernard Blettery, Medical ICU, Dijon university Hospital, Dijon, France.

Bernard Claud, General ICU, Le Puy en velay Hospital, Le Puy en velay, France.

Jean Yves Lefrant, General ICU, Nimes University Hospital, Nimes, France.

Jean Michel Arnal, Medical ICU, Toulon Hospital, Toulon, France.

Carole Ichai, Surgical ICU, Nice University Hospital, Nice, France.

Olivier Leroy, Genera ICU, Tourcoing Hospital, Tourcoing, France.

Benoît Valet, General ICU, University hospital of Lille, Lille, France.

Olivier Pajot, General ICU, Argenteuil Hospital, Argenteuil, France.

Bernard Garrigues, General ICU, Aix en provence Hospital, Aix-en-provence Hospital, France.
